# Detailed Characterization of Immune Cell Infiltrate and Expression of Immune Checkpoint Molecules PD-L1/CTLA-4 and MMR Proteins in Testicular Germ Cell Tumors Disclose Novel Disease Biomarkers

**DOI:** 10.3390/cancers11101535

**Published:** 2019-10-11

**Authors:** João Lobo, Ângelo Rodrigues, Rita Guimarães, Mariana Cantante, Paula Lopes, Joaquina Maurício, Jorge Oliveira, Carmen Jerónimo, Rui Henrique

**Affiliations:** 1 Department of Pathology, Portuguese Oncology Institute of Porto (IPOP), R. Dr. António Bernardino de Almeida, 4200-072 Porto, Portugal; jpedro.lobo@ipoporto.min-saude.pt (J.L.); angelorod57@gmail.com (Â.R.); ritaguimaraes.apct@gmail.com (R.G.); marianacantantecf@gmail.com (M.C.); lopesanapaula.s@gmail.com (P.L.); 2 Cancer Biology and Epigenetics Group, Research Center of Portuguese Oncology Institute of Porto (GEBC CI-IPOP) and Porto Comprehensive Cancer Center (P.CCC), R. Dr. António Bernardino de Almeida, 4200-072 Porto, Portugal; 3 Department of Pathology and Molecular Immunology, Institute of Biomedical Sciences Abel Salazar, University of Porto (ICBAS-UP), Rua Jorge Viterbo Ferreira 228, 4050-513 Porto, Portugal; 4 Department of Medical Oncology & Urology Clinic, Portuguese Oncology Institute of Porto (IPOP), R. Dr. António Bernardino de Almeida, 4200-072 Porto, Portugal; jmauricio@ipoporto.min-saude.pt; 5 Department of Urology & Urology Clinic, Portuguese Oncology Institute of Porto (IPOP), R. Dr. António Bernardino de Almeida, 4200-072 Porto, Portugal; urojorge@gmail.com

**Keywords:** CTLA-4, immune checkpoints, mismatch repair, PD-L1, prognosis, survival, testicular germ cell tumors

## Abstract

*Background:* The immune infiltrate plays an important part in testicular germ cell tumors, but it remains scarcely studied. We aimed at thoroughly characterizing the immune infiltrate and expression of immune checkpoints PD-L1/CTLA-4 and mismatch repair (MMR) proteins in these neoplasms, seeking for associations with patient outcome. *Methods:* A total of 162 consecutively diagnosed patients (2005–2018) were included. Immunostaining for PD-L1, CTLA-4 and MMR proteins was independently assessed both in immune cells (ICs) and tumor cells (TCs) of primary tumors and metastases, and characterization of IC populations was pursued. *Results:* PD-L1 and CTLA-4 positivity in ICs was frequent (85.5% and 96.3%). Patients with absent PD-L1 positive ICs exhibited significantly worse relapse-free survival (hazard ratio = 4.481, 95% CI 1.366–14.697, *p* = 0.013), both in univariable and multivariable analysis. Lower CD20 and CD3 IC infiltration in seminomas associated with higher disease stage (*p* = 0.0216, *p* = 0.0291). CTLA-4 TC intensity was significantly higher in yolk sac tumor, choriocarcinoma and teratoma, while PD-L1 TC positivity was significantly more frequent in choriocarcinoma. Both PD-L1 and CTLA-4 immunoexpression in ICs of metastatic samples was frequent (100% and 88.2%). MMR proteins were differentially expressed among the different tumor subtypes. *Conclusions:* Immune infiltrate/checkpoints associate with patients’ outcome, constituting novel (potentially targetable) disease biomarkers.

## 1. Introduction

Testicular germ cell tumors (TGCTs) are the most common cancer type afflicting Caucasian men between 15–44 years-old. The overall prognosis is good, with outstanding cure rates and decreasing mortality [1]. However, disease relapse and resistance to cisplatin constitute major clinical challenges [2,3]. Moreover, one should take into consideration the morbidity induced by chemo- and radiotherapy in such young patients [4,5]. Therefore, novel therapies showing improved antitumor activity, less toxicity and with the ability to overcome cisplatin resistance are desirable [6]. 

TGCTs are fascinating neoplasms, which relate to embryonic and germ cell development [7,8]. They exhibit striking heterogeneity, comprehending various tumor types, of which type II tumors are the most frequent and clinically relevant. These derive from a common precursor lesion, germ cell neoplasia *in situ* (GCNIS), and are grouped into two major families: the seminomas (SEs) and the various non-seminoma (NS) subtypes (embryonal carcinoma [EC], postpubertal-type yolk sac tumor [YST], choriocarcinoma [CH] and postpubertal-type teratoma [TE]) [9]. Given this diversity, it is fair to assume that the immune infiltrate present within these neoplasms might also be heterogeneous on its type and role. Indeed, one of the most well-known features of SEs is the presence of fibrous septa filled by lymphocytes. However, exceptions to this “classical” pattern are not infrequent, from evidence of true lymphoid follicles or epithelioid granulomas, to almost absence of immune cells, to the exquisite event of “burned-out” tumors [10,11,12,13].

Immunotherapies have achieved important landmarks with clinical impact over the last years in many cancer models, including urological cancer [14]. However, concerning TGCTs, the study of tumor microenvironment and development of immunotherapeutic strategies was only set in motion more recently [6,14]. In 2015, Fankhauser et al. first indicated programmed death receptor ligand 1 (PD-L1) as a promising therapeutic target in TGCTs, demonstrating its immunoexpression in tumor and stromal cells [15]. In an analysis of *The Cancer Genome Atlas* (TCGA) database, Shah et al. identified a surrogate signature of “T-cell inflamed genes” in 47% of TGCTs, and Siska et al. explored in depth the immune infiltrate in TGCTs [16,17]. Since then, both high PD-L1 immunoexpression in tumor cells (TCs) and low immunoexpression in immune cells (ICs) were found associated with poorer prognosis in two different studies [18,19]. Also, Hinsch et al. demonstrated frequent immunoexpression of TIGIT and PD-1 in SE and Wei et al. showed the influence of the specific immune landscape on PD-L1 expression [20,21]. Despite individual reports of patients responding to PD-L1 blocking [22], the role of other immune checkpoints such as cytotoxic T-lymphocyte-associated antigen (CTLA-4) remains largely elusive in TGCTs.

Recently, mismatch-repair (MMR) deficiency has been strongly associated to PD-L1 expression, namely in colorectal and endometrial cancers [23,24]. MMR-deficient neoplasms seem to be more immunogenic, entailing higher levels of PD-L1 expression. Moreover, an association between MMR-deficiency, microsatellite instability (MSI) and cisplatin resistance was documented in TGCTs [25]. Indeed, more differentiated, OCT3/4-negative TGCTs were shown to exhibit lower MMR proteins expression, hypothesizing that this might explain the lower sensitivity to cisplatin treatment displayed by those tumor subtypes [26]. 

Herein, we aim to evaluate and compare the immunoexpression of PD-L1, CTLA-4 and MMR proteins in a large and well characterized cohort of TGCTs, exploring their potential biological role and establishing important clinicopathological associations (namely impact on patients’ outcome). Furthermore, we aim to explore associations between abundance of specific IC populations and clinicopathological variables in a cohort of SE tumor samples.

## 2. Results

### 2.1. Immune Cells in Testicular Germ Cell Tumors

#### 2.1.1. Immune Checkpoints Expression—PD-L1 and CTLA-4

A detailed clinicopathological characterization of the TGCT cohort is depicted in Table 1. Detailed composition of mixed tumors is described in Supplementary Table S1. One patient was diagnosed with synchronous bilateral tumors and other patient showed metachronous tumors (the first being an SE, on the left; the second occurring six years later, another SE, on the right).

**Figure 1:**
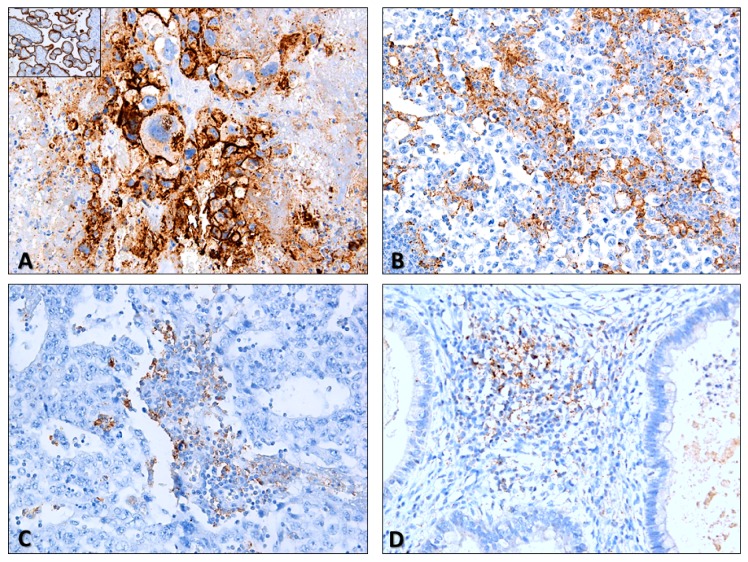
Illustrative examples of PD-L1 staining in testicular germ cell tumors. (**A**) Evident PD-L1 staining in a choriocarcinoma (brain metastasis). Notice the intense, clear membrane staining, in “chicken-wire” pattern (200×). *Inset:* positive control (placenta) for PD-L1 staining, included in all slides; (**B**) PD-L1 staining in immune cells (notice the granular, punctate staining pattern) in a pure seminoma. Some tumor cells also exhibited clear membrane staining (100×); (**C**) PD-L1 staining in immune cells in a pure embryonal carcinoma (200×); (**D**) PD-L1 staining in immune cells in a mixed tumor with teratoma components (200×).

**Figure 2:**
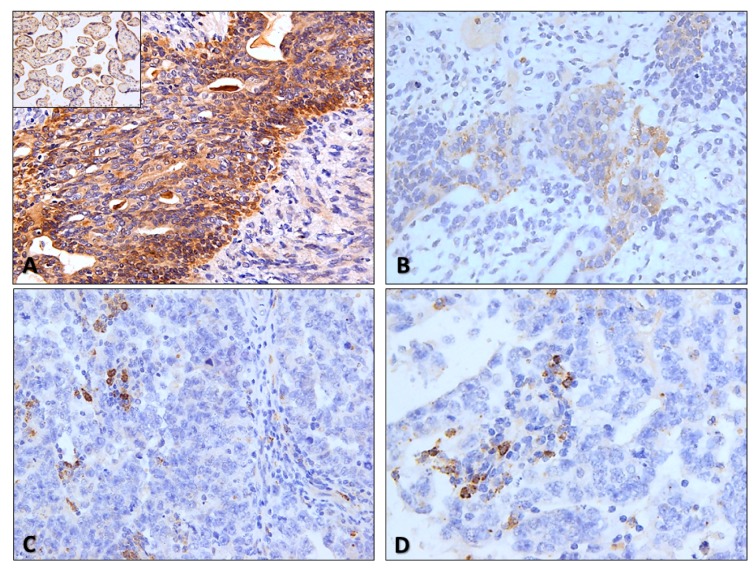
Illustrative examples of CTLA-4 staining in testicular germ cell tumors. (**A**) and (**B**) CTLA-4 strong (**A**) and moderate (**B**) intensity staining in tumor cells of residual mature teratomas in lymph node metastasis and in a primary tumor (immature teratoma component of a mixed tumor) (200×). Notice the predominantly cytoplasmic but also slight membrane staining. *Inset:* positive control (placenta) for CTLA-4 staining, included in all slides; (**C**) and (**D**) CTLA-4 staining in scattered immune cells populating two pure embryonal carcinomas (400×). Notice the punctate, granular staining, like for PD-L1.

**Figure 3:**
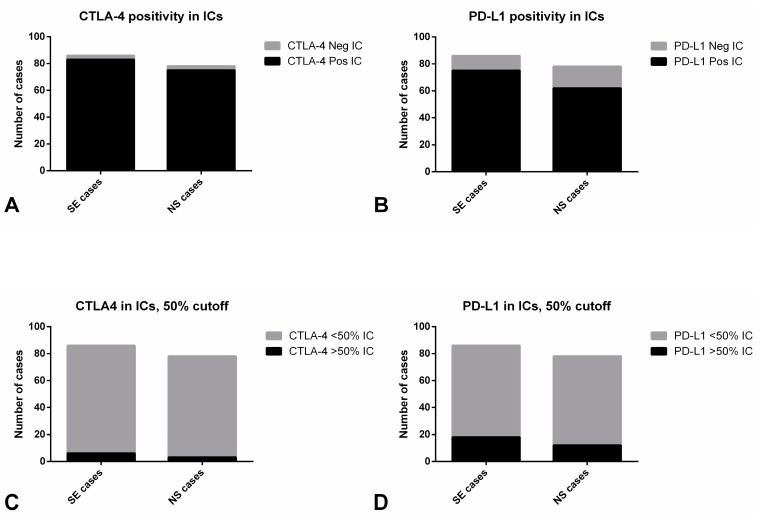
Immunoexpression of CTLA-4 and PD-L1 in immune cells of testicular germ cell tumors. CTLA-4 positivity (**A**) and expression above the 50% cutoff (**C**) in immune cells among seminomas and non-seminomas. PD-L1 positivity (**B**) and expression above the 50% cutoff (**D**) in immune cells among seminomas and non-seminomas. Abbreviations: IC—immune cells; NS—non-seminoma; SE—seminoma.

Distribution of PD-L1 and CTLA-4 immunoexpression in ICs is depicted in Table 2 and raw data is provided in Supplementary Table S3. Illustrative examples of immunostaining patterns for these markers are depicted in Figure 1 and Figure 2. PD-L1 and CTLA-4 positivity was overall observed in 137/158 (85.5%) and 158/164 (96.3%) of TGCTs. Immunoexpression invariably corresponded to high intensity, often punctate staining (Figure 1B–D and Figure 2C,D), hence we focused our analysis on the percent of positive ICs. There were no statistically significant differences in expression of both immune checkpoint molecules in ICs between SE and NS cases (Figure 3A,B), including when considering a 50% cutoff (Figure 3C,D).

CTLA-4 positivity in ICs did not associate with clinicopathological variables (Table 2). However, patients with CTLA-4 immunoexpression in ICs above the 50% cutoff exhibited significantly less lymphovascular invasion and presented with lower pT stage and N stage when compared to those with <50% positive ICs (*p* = 0.0345, *p* = 0.0419 and *p* = 0.0280, respectively, Supplementary Figure S1A–C, respectively). Positivity of CTLA-4 in ICs did not significantly impact relapse-free survival (RFS) (Supplementary Figure S2).

**Figure 4:**
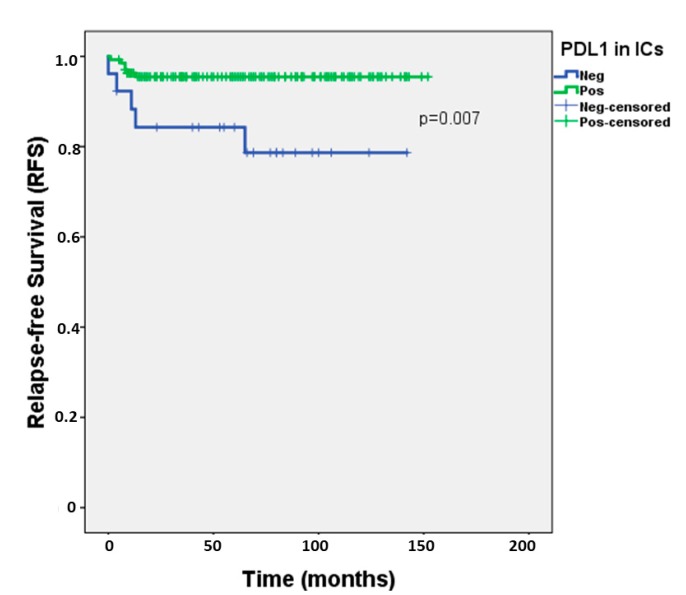
Relapse-free survival according to PD-L1 positivity in immune cells of testicular germ cell tumors. Abbreviations: IC—immune cells; RFS—relapse-free survival.

Regarding PD-L1 positivity in ICs, it did not differ significantly among subgroups of patients with different clinicopathological variables (lymphovascular invasion, TNM staging, presence of metastases or IGCCCG prognostic grouping) (Table 2). However, importantly, patients with absent PD-L1 positive ICs exhibited significantly worse RFS when compared to patients with presence of PD-L1 positive ICs (hazard ratio = 4.481, 95%confidence interval 1.366–14.697, *p* = 0.013) (Figure 4); 2-year-RFS for patients with presence and absence of PD-L1-positive ICs was of 95.5% and 84.3%, respectively. This was maintained in multivariable analysis, when adjusting individually for various clinicopathological variables (Table 3) and also when adjusting simultaneously for histology, TNM staging and lymphovascular invasion (hazard ratio = 3.874, 95% confidence interval 1.158-12.961, *p* = 0.028).

#### 2.1.2. Characterization of the Immune Infiltrate

Among the 86 pure SE patients, 43 (50.0%) and 59 (68.6%) were classified as being highly infiltrated with CD20- and CD3-positive ICs, respectively (specific criteria for considering IC infiltration are detailed in Methods section) (Supplementary Figure S3A,B). Lower CD20 IC infiltration associated with higher disease stage (*p* = 0.0216) and presence of *rete testis* invasion (*p* = 0.0079). Also, lower CD3 IC infiltration associated with higher disease stage (*p* = 0.0291) and pT stage (*p* = 0.0211) (Table 4). CD20 and CD3 IC infiltration in SE patients did not have a significant impact on RFS (*p* = 0.323 and *p* = 0.379, respectively).

Further characterizing the CD3-positive IC population, it consisted mostly of CD8-positive ICs; 61 (70.9%) and 80 (93.0%) SEs were regarded as highly infiltrated by CD4- and CD8-positive ICs. The CD4/CD8 ratio was <1 in 70 (81.4%) cases (Supplementary Figure S3C), with dominance of CD4-positive ICs in only 16 cases (18.6%). High infiltration by CD68 ICs was depicted in 49 (57.0%) cases (Supplementary Figure S3D). CD56-positive ICs were absent in 36 (41.9%) cases and present very focally in 50 (58.1%) cases (Supplementary Figure S3E).

Illustrative examples of different types of immune infiltrate are depicted in Supplementary Figure S4. 

### 2.2. Tumor Cells in Testicular Germ Cell Tumors and in Metastases

#### 2.2.1. Immune Checkpoints Expression in Primary Tumors—PD-L1 and CTLA-4

**Figure 5:**
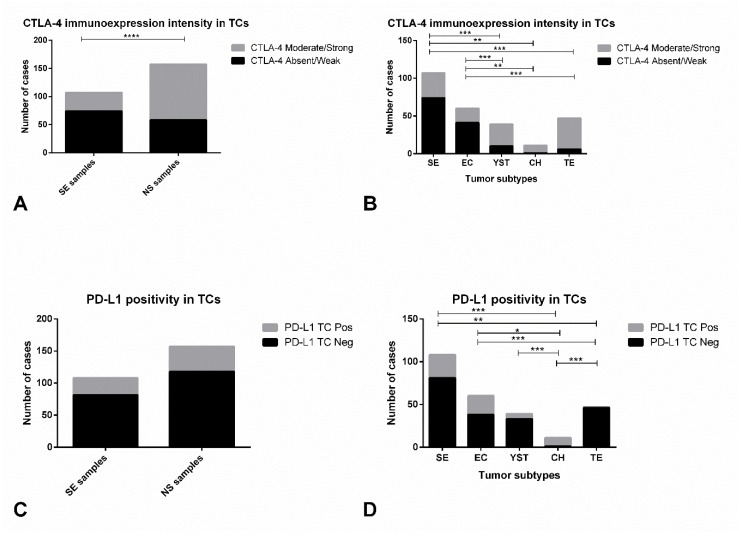
Immunoexpression of CTLA-4 and PD-L1 in tumor cells of testicular germ cell tumor subtypes. CTLA-4 immunoexpression intensity in tumor cells among seminomas and non-seminomas (**A**) and among the various histotypes (**B**). PD-L1 positivity in tumor cells among seminomas and non-seminomas (**C**) and among the various histotypes (**D**). Abbreviations: TC—tumor cells; NS—non-seminoma; SE—seminoma; EC—embryonal carcinoma; YST—postpubertal-type yolk sac tumor; TE—postpubertal-type teratoma; CH—choriocarcinoma. * refers to *p* < 0.05; ** refers to *p* < 0.01; *** refers to *p* < 0.001; **** refers to *p* < 0.0001.

Immunoexpression of CTLA-4 in TCs was depicted in 237/264 (89.7%) of available tumor samples; it is further detailed in Table 5 and illustrative examples are depicted in Figure 2A,B and Supplementary Figure S5. Staining was rather diffuse across the various subtypes, staining all available TCs; hence we focused our analysis on the intensity of staining (specific criteria indicated in Methods). Indeed, CTLA-4 immunoexpression was significantly more intense in NS when compared to SE (*p* < 0.0001, Figure 5A). Additionally, there were significant differences in immunoexpression intensity of CTLA-4 in TCs of the various tumor subtypes (*p* < 0.0001); specifically, it was more intense in YST, CH and TE, when compared to EC or SE (Figure 5B).

There were no significant associations between the intensity of CTLA-4 immunoexpression in TCs and the various clinicopathological variables (staging, IGCCCG grouping, lymphovascular invasion or *rete testis* invasion, pT stage or N stage). CTLA-4 TC intensity did not have a significant impact on RFS (*p* = 0.934) (Supplementary Figure S6). 

PD-L1 immunoexpression in TCs (Table 5) was observed in 66/265 (24.9%) tumor samples. Illustrative examples are depicted in Figure 1A,B. Its intensity was rather similar among subtypes, hence we focused on the proportion of positive TCs. Overall, the proportion of PD-L1-positive cases did not differ significantly between SE and NS (Figure 5C). However, there were significant differences in PD-L1 immunoexpression among the various subtypes of TCs (*p* < 0.0001); specifically, PD-L1 positivity in TCs was significantly more common in CH and less common in TE (Figure 5D).

**Figure 6:**
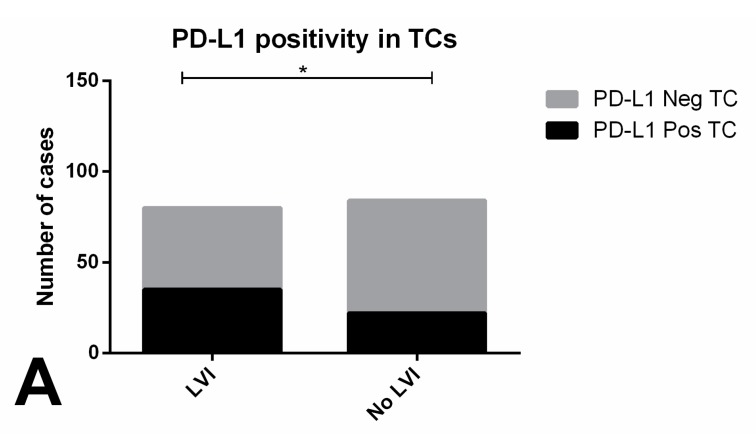

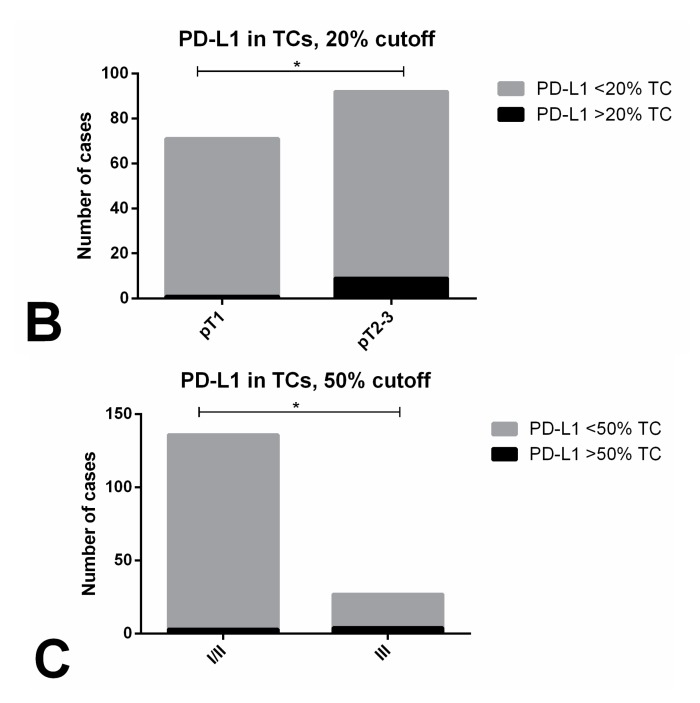
Immunoexpression of PD-L1 in tumor cells of testicular germ cell tumor subtypes and association with clinicopathological features. (**A**) association between PD-L1 positivity and lymphovascular invasion; (**B**) association between PD-L1 positivity above the 20% cutoff and pT stage; **C**) association between PD-L1 positivity above the 50% positivity and TNM staging. Abbreviations: TC—tumor cells; LVI—lymphovascular invasion. * refers to *p* < 0.05.

Patients with evidence of PD-L1-positive TCs showed significantly more frequent lymphovascular invasion compared to those with no PD-L1 immunoexpression in TCs (*p* = 0.0219, Figure 6A). PD-L1 staining in >20% and >50% TCs associated with pT2-3 stage and with stage III disease, respectively (*p* = 0.0439, *p* = 0.0150) (Figure 6B,C respectively). There were no significant associations with the remaining clinicopathological variables. Also, RFS did not differ significantly among patients showing PD-L1-positive and negative TCs (*p* = 0.846) (Supplementary Figure S7).

#### 2.2.2. Mismatch Repair Protein Expression in Primary Tumors

**Figure 7:**
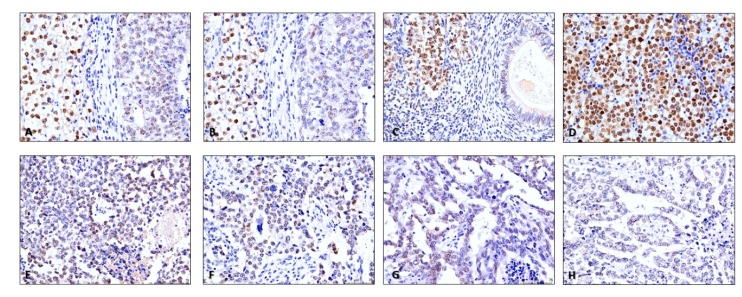
Illustrative examples of mismatch repair proteins staining in testicular germ cell tumors. (**A**) and (**B**)—MLH1 (**A**) and PMS2 (**B**) staining patterns in a mixed tumor composed of seminoma (on the left) and embryonal carcinoma (on the right). Notice the clearly weaker intensity staining in embryonal carcinoma and reduced number of stained cells when compared to the seminoma component (200×); **C**—MSH2 staining in a mixed tumor composed of seminoma (on the left) and teratoma (on the right). Notice the clearly stronger intensity staining in seminoma when compared to the teratoma element (200×); **D**—MLH1 strong and diffuse intensity staining in a pure seminoma. This was the most common pattern witnessed in pure seminomas (200×); **E**–**H**—MSH2 (**E**), MSH6 (**F**), MLH1 (**G**) and PMS2 (**H**) staining in the same post-chemotherapy metastatic lung metastasis, composed of embryonal carcinoma. The patient was refractory to multiple courses of cisplatin. Notice the rather weak staining and reduced number of stained cells, rendering a low immunoscore (200×).

Immunoexpression of MMR proteins in primary tumors is depicted in Table 5 and illustrative examples are depicted in Figure 7A–D. Both immunoexpression intensity and proportion of positive cells were variable across the various TGCT cases, and so a combined score was considered (as mentioned in the Methods section). 

**Figure 8:**
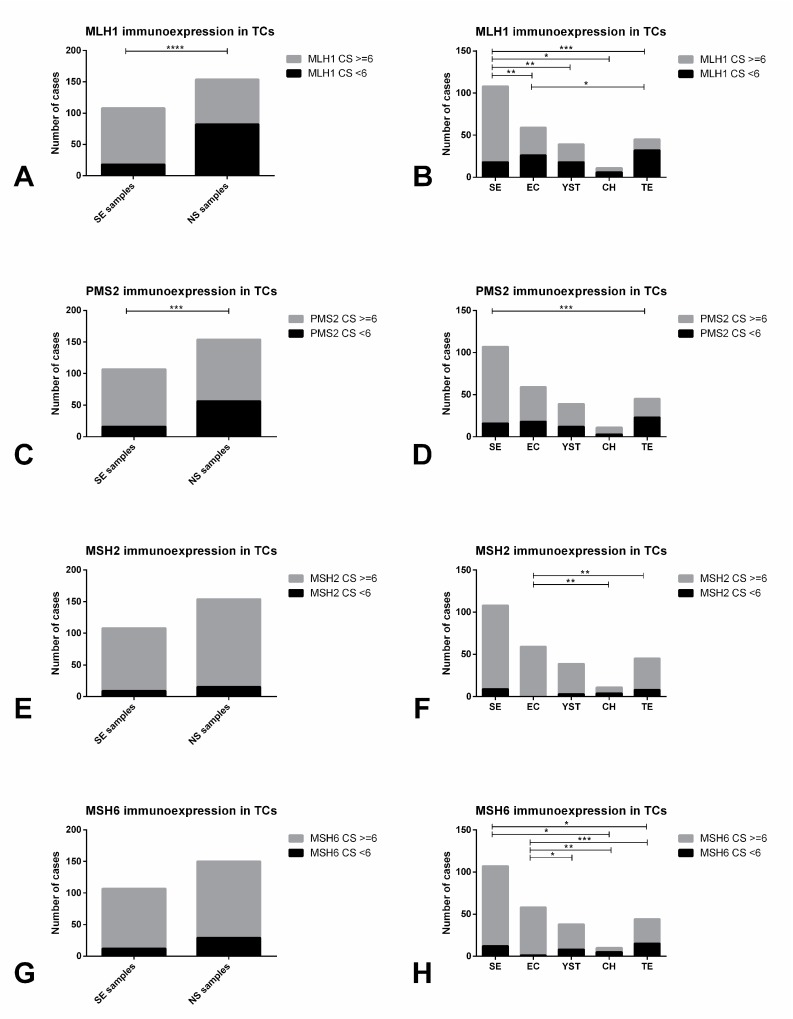
Immunoexpression of mismatch repair proteins in tumor cells of testicular germ cell tumor subtypes. MLH1 (**A**) and (**B**), PMS2 (**C**) and (**D**), MSH2 (**E**) and (**F**) and MSH6 (**G**) and (**H**) immunoexpression scoring in tumor cells among seminomas and non-seminomas, and among the various histotypes. Abbreviations: CS—combined score; TC—tumor cells; NS—non-seminoma; SE—seminoma; EC—embryonal carcinoma; YST—postpubertal-type yolk sac tumor; TE—postpubertal-type teratoma; CH—choriocarcinoma. * refers to *p* < 0.05; ** refers to *p* < 0.01; *** refers to *p* < 0.001; **** refers to *p* < 0.0001.

SEs exhibited significantly higher immunoexpression scores for MLH1 and PMS2 when compared to NS (*p* < 0.0001 and *p* = 0.0003, respectively, Figure 8A,C). There were also significant differences in the immunoexpression scores of all four MMR proteins among the various TGCT subtypes (*p* < 0.0001, *p* = 0.0003, *p* = 0.0005 and *p* < 0.0001 for MLH1, PMS2, MSH2 and MSH6, respectively). A combined score ≥6 was significantly more common in SE when compared to all other subtypes (for MLH1) and when compared to TE (for PMS2) (Figure 8B,D). It was also significantly more frequent in EC when compared to CH and TE (for MSH2) and when compared to all other subtypes (for MSH6) (Figure 8E–H). Remarkably, MSH2 and MSH6 combined score was ≥6 in most samples in all different tumor subtypes.

We found no significant association between the combined immunoexpression score for any of the MMR proteins and the presence of PD-L1-positive TCs or ICs. For SEs, also no association was found between immunoexpression score of any of the four MMR proteins and the infiltration by CD20- or CD3-positive ICs.

#### 2.2.3. Immunoexpression of the Several Markers in TGCT Metastases

Raw data regarding immunoscoring of metastatic samples is provided in Supplementary Table S4. CTLA-4 immunoexpression in ICs and TCs in metastatic samples was found in 15/17 (88.2%) and 14/17 (82.4%) of the cases. The proportion of positive ICs and the presence or intensity of CTLA-4 staining in TCs did not differ significantly between primary tumors and metastases (Supplementary Figure S8A,B). 

PD-L1 immunoexpression in ICs and TCs was observed in 17/17 (100%) and 8/17 (47.1%) of available metastatic samples, both with higher frequency when compared to primary TGCT samples, although the difference was not statistically significant (*p* = 0.0801 and *p* = 0.0827) (Supplementary Figure S8C,D). Positivity for PD-L1 in ICs was seen in 1–10% of cells in 8 samples, in 10–20% of cells in 6 samples, in 20–30% of cells in 2 samples and in 50–60% of cells in 1 sample.

**Figure 9:**
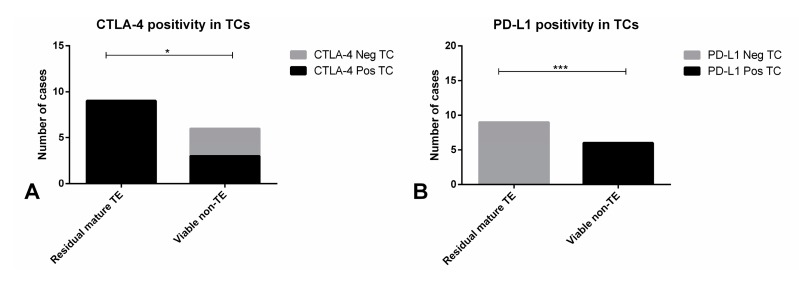
Immunoexpression of CTLA-4 and PD-L1 in tumor cells of metastatic testicular germ cell tumor samples. Distribution of CTLA-4 positivity (**A**) and PD-L1 positivity (**B**) in tumor cells among residual mature teratoma and non-teratoma viable disease. Abbreviations: TC—tumor cells; TE—teratoma. * refers to *p* < 0.05; *** refers to *p* < 0.001.

All nine residual mature TE samples disclosed immunoexpression of CTLA-4 in TCs, while this was observed in 3/6 (50%) of non-TE viable disease (*p* = 0.0440, Figure 9A). Importantly, all six metastatic samples with cisplatin-resistant viable non-TE disease showed positivity in TCs for PD-L1, but none of the nine residual mature TE masses showed PD-L1-positive TCs (*p* = 0.0002, Figure 9B). Illustrative examples of MMR proteins immunoexpression in metastatic samples are depicted in Figure 7E–H. Low immunoexpression scores for the various MMR proteins were overall more frequent in residual mature TE cases (5/9, 55.6% for MLH1; 8/9, 88.9% for PMS2; 6/9, 66.7% for MSH2; and 4/9, 44.4% for MSH6) when compared to non-TE viable disease (2/6, 33.3% for MLH1 and PMS2; 3/3, 50% for MSH2; and 1/4, 25% for MSH6), but the difference did not reach statistical significance (Supplementary Figure S9A–D, respectively).

## 3. Discussion

Better prognostic biomarkers and also novel, less toxic, treatment regimens are needed for TGCT patients. The ability to avoid immune destruction has been established as one of the hallmarks of cancer [28] and immunotherapies have proved their value in various neoplasms, including urological cancers [14] such as urothelial and kidney cancer [29,30]. Despite the often abundant immune infiltrate that populates TGCTs, data on immune checkpoints expression and on TGCT microenvironment is still scarce.

Remarkably, we found that the great majority of TGCTs exhibited CTLA-4 and PD-L1 positivity in infiltrating ICs (96.3% and 85.5%), and that this occurred across all subtypes. Higher PD-L1 immunoexpression scoring has already been indicated to associate with a better outcome in urothelial carcinoma, for which PD-L1 scoring in ICs has become routine in selected groups of patients [31]. The only work fully dedicated to this matter on TGCTs found PD-L1 immunoexpression in ICs occurring in 95.9% of SEs (higher frequency when comparing to our study, where 87.2% SEs evidenced PD-L1 IC staining) and that lower PD-L1 immunoexpression in ICs was associated with various adverse clinicopathological features and with worse progression-free survival [19]. Direct comparison with our findings must be done with caution given the various methodological differences between the studies: cohort size, use of tissue microarrays vs. whole slide interpretation, method of PD-L1 scoring —proportion of positive cells vs. specific scoring method—and, importantly, distinct anti-PD-L1 clones, which are largely known to affect the interpretation of staining [32]. Also, different protocols used for antigenic recovery might influence the readout of immunoscoring. Nevertheless, our results are overall in line with the previous observations. Importantly, we have showed that PD-L1 immunoexpression in ICs had an independent impact on RFS when adjusting for several other clinical variables (individually or as a group); this has been shown before only when adjusting for IGCCCG grouping alone [19]. Indeed, PD-L1 has a major role in modulating T-cell activation and the phenomena of immune tolerance; hence, we and others [19] hypothesize that a decrease or even absence of PD-L1-positive ICs might facilitate tumor dissemination or impair response to treatment. Curiously, the same pattern was found for CTLA-4 immunoexpression in ICs, with higher expression levels associating with good prognostic features such as less lymphovascular invasion and lower pT and N stage. CTLA-4 is also involved in the regulation of T-cell activation, self-tolerance and regulation of T-cell function, chronic inflammation and anti-tumor activity [33]. However, molecular mechanisms are still to be better elucidated, but it might be possible that, as for PD-L1, lower expression levels might interfere with response to treatment or ease to proliferate and spread [34]. 

Given the prominent immune infiltrate across TGCT subtypes, it is surprising that few studies have attempted to better study this immune infiltrate. Previous works have been limited in cohort size and heterogeneity; some have focused on male fertility, comparing to biopsies of infertile patients or those with GCNIS; and, importantly, there is an absence of studies seeking for clinicopathological correlates, which is what we pursued in our work [35,36,37,38,39,40,41]. Remarkably, both tumors with low B-cell and low T-cell infiltration were found to associate with aggressive prognostic features, including stage. This finding puts in evidence the anti-tumor effect of the immune infiltrate in these tumors. Older studies have showed conflicting results regarding the CD4/CD8 T-cell predominance in SEs [35,37]. However, these have been very limited in size (20 and 27 patients). Our results in 86 patients showed that most T-lymphocytes present within SEs are CD8+ cytotoxic T-lymphocytes, which is in line with the more recent cytotoxic environment assumed to be present in SEs, contributing to high apoptosis and to the excellent prognosis of this tumor subtype [38,42]. 

PD-L1 immunoexpression has been documented in TCs of several neoplasms, including in non-small cell lung carcinomas for which its assessment has become a routine procedure in pathology laboratories [43]. It has also been demonstrated in TGCTs [15] and associated with poor prognostic features [18]. In our study, we found an overall positivity for PD-L1 in TCs in 24.9% tumor samples (25% of SEs and 24.8% of NS). Our findings differ from those of two previous studies, which showed a higher frequency of PD-L1 staining (73–76% in SEs and 64–89% in NS). Again, comparisons should be cautiously done: studies used different anti-PD-L1 clones—in our study we have used the 22C3 Dako antibody, in the same system approved for routine diagnostic use in our Institution in lung cancer, while the aforementioned previous studies used clones E1L3N (Cell Signaling) and Abcam (EPR1161(2): AB174838); assessed PD-L1 positivity in both TCs and stromal cells together, while we only considered TCs; used tissue-microarrays, while we evaluated the whole representative slide; considered non-membrane staining in TCs as positive, while we followed guidelines reported for non-small cell lung carcinoma, considering only membrane staining of any intensity; used diverse multiplicative scores, while we reported the proportion of positive TCs, again as recommended in non-small cell lung cancer. All these differences in methodology (especially in interpretation of positive staining, which was much more restrictive and similar to current guidelines for immunotherapy response prediction in our study) might have impact in conclusions. PD-L1 expression in both previous studies was found to be variable among subtypes, a finding we too confirmed. In line with these works, we also found PD-L1 immunoexpression to be the highest in CH (which is not surprising, since this tumor subtype resembles placental tissue, which is precisely one of the most universal positive control samples used for PD-L1 testing, related to the need for regulating immune response against the growing fetus—the fetomaternal tolerance [44]) followed by EC and SE, and lowest in YST and TE [15]. Moreover, as in the previous work [18], we too corroborated the association of high PD-L1 scoring in TCs with poor prognostic features, including disease stage. CTLA-4 immunoexpression in tumor cells has been quite controversial in literature, as has been choice of antibody for this protein, despite some reports with positive findings [45]. In our exploratory study, preliminary data indicated that expression occurred frequently in TGCTs (89.7% of the tumor samples). Also, it was significantly more intense in NS cases. This high frequency of positivity for this immune checkpoint molecule could potentially indicate that anti-CTLA-4 targeted therapies might be useful in TGCTs, although this should be interpreted with caution as until now CTLA-4 expression has not been validated as a biomarker of response to targeted therapy. Also, given the limitations of immunohistochemistry for assessing CTLA-4 expression and controversy related to its use, future studies are surely needed to validate such findings. We have therefore performed an *in silico* analysis of the TCGA database (*n* = 156 TGCT samples [46]), which revealed mRNA expression of this target throughout the cohort (Supplementary Figure S10). Immunoexpression was significantly higher in CH (like for PD-L1, again reflecting the immune tolerance microenvironment in the placenta [47]) but also in YST and TE, in which PD-L1 expression was infrequent.

To date, no assessment of either PD-L1 or CTLA-4 was reported in TGCT metastatic samples. Our findings show frequent immunoexpression (both in TCs and ICs) of PD-L1 and CTLA-4 in metastatic samples. Specifically, for PD-L1, immunoexpression was more frequent in metastases than in primary tumors (including positive ICs in 100% of the cases). Remarkably, all metastatic samples with non-TE cisplatin-resistant viable disease showed PD-L1 positivity in TCs. This could potentially indicate that drugs targeting these agents could be of use in the metastatic setting. However, importantly, data should be interpreted with caution given the failure of clinical studies employing targeted therapies as single agents thus far [48,49,50]. Also, despite constituting the largest series thus far with immunoexpression scoring of immune checkpoints in 17 metastatic samples, still the total number of tumors is modest. 

Despite some controversy among initial studies (with small series) [51], the association between MMR-deficiency, MSI and cisplatin resistance was finally confirmed in larger studies [25]. Additionally, we have recently described the first case of a TGCT (a seminoma patient) directly attributed to a MSH2-mutated Lynch syndrome, further expanding the spectrum of the disease [52]. MMR-deficiency and subsequent genomic instability trigger formation of neoantigens and immunogenicity, so that MMR-deficiency frequently predicts a favorable response to anti-PD-L1 agents. However, in our work, we found no significant association between the immunoexpression score for MMR proteins and the amount of B- or T-lymphocytes, or the expression of PD-L1, which seems to indicate that the immune infiltration and expression of immune checkpoints is at least not completely explained by this mechanism (perhaps implying failure of other DNA repair mechanisms like the homologous recombination system, as genes involved in this pathway were found to be frequently silenced in TGCTs [53]). This requires further validation in larger series. MMR protein expression significantly differed among the various tumor subtypes, reflecting and further extending data of previous works [54]. Velasco et al. [54] showed that SEs less frequently exhibited low MSH2 scores, and that TE and CH showed more frequently a low MLH1 and MSH2 score. Our findings are in line with these data. It is reported that more differentiated OCT3/4-negative TGCTs frequently exhibit lower MMR proteins expression, potentially contributing to lower cisplatin sensitivity in these subtypes. Our data precisely indicate that MMR scores are significantly higher in SE and EC (OCT3/4-positive, more undifferentiated subtypes) compared to YST, CH and TE (OCT3/4-negative, more differentiated subtypes). Also, this occurred in the metastatic context, as immunoexpression scores were lower in residual mature TE when compared to non-TE metastases. Additionally, the pair MSH2 and MSH6 in our cohort showed very frequently high immunoexpression scores across all primary tumor subtypes, while for the pair MLH1 and PMS2 differences among histologies were more remarkable, perhaps suggesting that the latter pair is more directly related to this progressive differentiation and loss of OCT3/4 expression.

A recent report found that methylation of homologous recombinant genes was associated with the expression of the immune checkpoint PD-L1 in squamous cell carcinoma of the head and neck, lung and cervix [55], and promoter methylation of homologous recombination-related genes was found to be frequent in TGCTs [53]. As such, the methylation status of these genes could represent a new predictive biomarker for immune checkpoint inhibition, a strategy we wish to further explore in future studies.

Limitations of our work include its retrospective nature, the subjectivity inherent to immunohistochemistry scoring, and the modest number of metastatic samples available in the study. Further studies are needed to replicate our findings and validate them, particularly in the metastatic context.

## 4. Materials and Methods

### 4.1. Patients and Samples

A cohort of TGCT patients (already described and validated by us [27]) consecutively diagnosed at the Portuguese Oncology Institute of Porto between 2005 and 2018 were included in the study. A total of 162 GCNIS-related TGCT patients were included. All patients were diagnosed and treated at this Institution by the same multidisciplinary team. Clinical files and all histological data were reviewed according to the most recent 8^th^ edition of the American Joint Committee on Cancer (AJCC) staging manual [56] and the 2016 World Health Organization (WHO) Classification of Tumours of the Urinary System and Male Genital Organs, respectively [9]. Patients with metastatic disease were further categorized according to the International Germ Cell Cancer Collaborative Group (IGCCCG) prognostic system [57,58]. Follow-up was last updated on May 2019.

Formalin-fixed paraffin-embedded (FFPE) orchiectomy tissue samples (prior to any systemic treatment) and metastases (lymph-node and/or visceral metastases) were available, and representative blocks (with >80% tumor cellularity, at least 1 cm^2^ of tissue and absence of extensive necrosis) were selected by a TGCT-dedicated pathologist. Consultation cases, cases corresponding to testicular type I or III tumors (prepubertal-type tumors and spermatocytic tumors), and cases without adequate histological material available were excluded. Importantly, individual tumor areas were carefully demarcated (in a strategy already reported by us [59,60,61]) and each component present in mixed tumors was independently considered for immunoexpression evaluation. In this line, a total of 271 independent tumor samples (109 SE, 61 EC, 41 YST, 12 CH and 48 TE) were included in the study (individual components of mixed tumors are detailed in Supplementary Table S1). Of the 17 metastases (15 after chemotherapy, two chemo naïve) with available biological material (patients who underwent retroperitoneal lymph-node dissection [RPLND] or metastasis resection), six constituted (histologically proven) cisplatin resistant disease in the form of viable non-TE elements. From all these cases, five micrometer sections were ordered for immunohistochemistry.

This study was approved by the ethics committee of Portuguese Oncology Institute of Porto (Comissão de Ética para a Saúde—CES-IPO-3-2018) and is within the scope of the project also approved by the same ethics committee (Comissão de Ética para a Saúde—CES-IPO-1-2018).

### 4.2. Immunohistochemistry

The automated staining instruments, detection systems, antibodies, dilutions, respective clones and vendors used in the study are depicted in Supplementary Table S2. Appropriate positive controls were used: human normal placenta and tonsil for PD-L1 and CTLA-4; normal colon mucosa for MLH1, MSH2, MSH6 and PMS2; normal pancreas (with Langerhans islets) for CD56; lymph node with abundant macrophages for CD68; and normal tonsil for the remainder. 

Immunostaining was assessed by a TGCT-dedicated pathologist with experience in routine reporting of PD-L1 immunoexpression in non-small cell lung cancer and urothelial carcinoma, blinded to clinicopathological data. For all the markers studied, no specific cutoff or methodology is validated for scoring immunoexpression. Hence, for the immune checkpoints PD-L1 and CTLA-4, both intensity (from 0 to 3—absent, weak, moderate and strong) and percentage of positive cells (with 10% increments) were scored, both for tumor cells (TCs) and for immune cells (ICs). “Weak” was defined as slight staining only depicted at high power magnification (400×); “moderate” as staining discernible at low power magnification (40×) but better appreciated and characterized at 100× magnification; and “strong” as staining optimally and easily appreciated at low power magnification (40×). For PD-L1 evaluation in TCs, the same indications reported for use in non-small cell lung cancer were employed, with only membrane staining (partial or complete) of any intensity being considered [62]. A minimum of 100 viable tumor cells were required to perform the evaluation, resulting in exclusion of cases that did not comply to this rule in the available stained tissue section. For evaluation in ICs, any membrane and/or cytoplasmic staining, often punctate, of mononuclear inflammatory cells (lymphocytes and macrophages) within tumor nests and/or immediately adjacent supporting stroma (convincingly associated with tumor response) was considered, such as recommended for urothelial carcinoma [63]. For CTLA-4, the same methodology reported by Paulsen and co-workers was used [45]. Briefly, membrane and/or cytoplasmic staining (separately in TCs and ICs) was annotated. ICs often showed the same punctate pattern exhibited upon PD-L1 staining. 

In all pure SE patients, the immune infiltrate within and at the periphery of the tumor was further characterized with CD20 (for B-lymphocytes), CD3 (for T-lymphocytes), CD4 (for T-helper lymphocytes), CD8 (for T-cytotoxic lymphocytes), CD68 (for macrophages) and CD56 (for NK cells). Cases were regarded as “highly” or “poorly” infiltrated by these cells in low-power assessment (proportion of available positive ICs in relation to the amount of viable TCs on the slide). “High infiltration” was defined as presence of true large follicle-shaped aggregates of immune cells throughout the tumor or completely filled septa overgrowing tumor cells, while “low infiltration” was considered when immune cells were found scattered throughout the tumor in septa, with only minor and few aggregate formation. To evaluate the predominance of each subtype of T-lymphocyte, the CD4/CD8 ratio was assessed. 

For MMR proteins, again, both intensity (from 0 to 3—absent, weak, moderate and strong) and proportion of positive TCs (in 25% increments, each category being attributed 1 point) was scored. A combined score resulting of the sum of both intensity and percentage scores was calculated.

In mixed tumor patients, the highest immunoexpression score for each of the markers was chosen as representative of the whole tumor (and the patient) for seeking clinicopathological correlates.

### 4.3. Statistical Analysis

Data was tabulated using Microsoft Excel 2016 and analyzed using IBM SPSS Statistics version 24 and GraphPad Prism 6. Associations between categorical variables were established by the Chi-square or Fisher’s exact test, as appropriate. Differences between distributions of continuous variables among groups were assessed by non-parametric Mann-Whitney U test and Kruskal-Wallis test, as appropriate. All *p*-values were adjusted to multiple testing by Bonferroni’s correction. Survival analyses were computed with Kaplan-Meier estimator and log-rank test. Relapse-free survival was assessed and hazard ratios with respective 95% confidence intervals were estimated using Cox regression models. Statistical significance was set at *p*-value < 0.05.

## 5. Conclusions

To conclude, we have demonstrated an overall frequent expression of immune checkpoints in TGCTs, both primary and metastatic, and showed that heterogeneity in expression levels of these markers and variable amount and types of immune infiltrate associate with prognosis. Importantly, PD-L1 immunoexpression in ICs influenced patient outcome, when adjusting for various relevant clinicopathological features. To the best of our knowledge, expression of CTLA-4 in TGCTs has not been reported to date; our data should be validated in independent series and by techniques other than immunohistochemistry in future studies. Also, we have demonstrated differential expression of MMR proteins among tumor samples, both in primary and metastatic context, possibly indicating association with differentiation and resistance to chemotherapy, although validation in larger cohorts is warranted. Despite failure of recent clinical studies using targeted therapies against these proteins as single agents, they were performed in unselected populations. Also, there are preliminary results indicative of some clinical activity for the combination of durvalumab and tremelimumab [49]. Given their epigenetic background, epigenetic drugs targeting DNA/histone methyltransferases or histone deacetylases could be promising partners to combine with immunotherapies and help overcome cisplatin resistance [2,64,65].

Overall, the immunobiology of (testicular) germ cell tumors remains an understudied field [66], and the key to achieve sound clinical effects might reside in uncovering the most relevant biomarkers (both histological, genetic or epigenetic) that might really indicate a favorable response to immunotherapies [67].

## Figures and Tables

**Table 1:** Clinicopathological features of testicular germ cell tumor patients.

Variables	Patient Cohort (*n* = 162 ^#^) Tumor Samples (*n* = 271)
Age [years (median, IQR)]	30 (25–36)
Laterality (*n*, %)	
Right	88/160 (55.0)
Left	71/160 (44.4%)
Bilateral synchronous	1/160 (0.6%)
Pre-operative AFP (*n*, %)	
Within normal range	102/157 (65.0)
Elevated	55/157 (35.0)
Pre-operative HCG (*n*, %)	
Within normal range	77/158 (48.7)
Elevated	81/158 (51.3)
Pre-operative LDH (*n*, %)	
Within normal range	75/131 (57.3)
Elevated	56/131 (42.7)
Histologic subtypes (*n*, %)	
Pure SE	86/164 (52.4)
Pure EC	12/164 (7.3)
Pure TE	4/164 (2.5)
Mixed tumor	62/164 (37.8)
Tumor components (*n*, %)	
SE	109/271 (40.2)
EC	61/271 (22.5)
YST	41/271 (15.1)
CH	12/271 (4.5)
TE	48/271 (17.7)
Largest tumor size [cm (median, IQR)]	4.2 (2.4-6.5)
*Rete testis* invasion (*n*, %)	
Absent	76/160 (47.5)
Present, stromal	84/160 (52.5)
Vascular invasion (*n*, %)	
Absent	84/164 (51.2)
Present	80/164 (48.8)
Stage (*n*, %)	
I	102/163 (62.5)
II	34/163 (20.9)
III	27/163 (16.6)
IGCCCG group* (*n*, %)	
Good	45/61 (73.8)
Intermediate	9/61 (14.7)
Poor	7/61 (11.5)
Histology of metastases (*n*, %)	
SE	1/17 (5.9)
EC	5/17 (29.4)
YST	1/17 (5.9)
CH	1/17 (5.9)
Residual mature TE	9/17 (52.9)
Relapse (*n*, %)	
No	151/162 (93.2)
Yes	11/162 (6.8)
Treatments performed (*n*)	
CT	102
RT	47
Vital status at last follow-up (*n*, %)	
A-NED	155/162 (95.7)
AWD	3/162 (1.9)
D-NED	1/162 (0.5)
DFD	3/162 (1.9)

^#^ Two patients had bilateral tumors. * For patients presenting with metastatic disease only. Abbreviations: A-NED—alive with no evidence of disease; AWD—alive with disease; CH—choriocarcinoma; CT—chemotherapy; DFD—died from disease; D-NED—died with no evidence of disease; EC—embryonal carcinoma; IGCCCG—International Germ Cell Cancer Collaborative Group; IQR—interquartile range; RT—radiotherapy; SE—seminoma; TE—postpubertal-type teratoma; YST—postpubertal-type yolk sac tumor. Exhaustive description of the cohort is depicted in [27].

**Table 2:** Association between CTLA-4 and PD-L1 immunoexpression in immune cells and clinicopathological features.

Variables	CTLA-4 in ICs: Negative	CTLA-4 in ICs: Positive	*p*-Value
Histology (*n*, % within Histology)			1.0
SE (*n* = 86)	3 (3.5)	83 (96.5)	
NS (*n* = 78)	3 (3.8)	75 (96.2)	
LVI (*n*, % within LVI)			0.6822
Yes (*n* = 80)	2 (2.5)	78 (97.5)	
No (*n* = 84)	4 (4.8)	80 (95.2)	
pT Stage (*n*, % within pT Stage)			1.0
pT1 (*n* = 71)	2 (2.8)	69 (97.2)	
pT2-3 (*n* = 92)	3 (3.3)	89 (96.7)	
N Stage (n, % within N Stage)			0.6599
N0 (*n* = 106)	4 (3.8)	102 (96.2)	
N+ (*n* = 56)	1 (1.8)	55 (98.2)	
Stage, TNM (*n*, % within Stage TNM)			0.6514
I (*n* = 102)	4 (3.9)	98 (96.1)	
II–III (*n* = 61)	1 (1.6)	60 (98.4)	
IGCCCG grouping (n, % within IGCCCG)			1.0
Good (*n* = 45)	1 (2.2)	44 (97.8)	
Intermediate/Poor (*n* = 16)	0 (0)	16 (100)	
	**PD-L1 in ICs: Negative**	**PD-L1 in ICs: Positive**	***p*-Value**
Histology (*n*, % within Histology)			0.2099
SE (*n* = 86)	11 (12.8)	75 (87.2)	
NS (*n* = 78)	16 (20.5)	62 (79.5)	
LVI (n, % within LVI)			0.0939
Yes (*n* = 80)	9 (11.3)	71 (88.7)	
No (*n* = 84)	18 (21.4)	66 (78.6)	
pT Stage (*n*, % within pT Stage)			0.1334
pT1 (*n* = 71)	15 (21.1)	56 (78.9)	
pT2-3 (*n* = 92)	11 (12.0)	81 (88.0)	
N Stage (*n*, % within N Stage)			0.5020
N0 (*n* = 106)	18 (17.0)	88 (83.0)	
N+ (*n* = 56)	7 (12.5)	49 (87.5)	
Stage, TNM (*n*, % within Stage TNM)			1.0
I (*n* = 102)	16 (15.7)	86 (84.3)	
II–III (*n* = 61)	10 (16.4)	51 (83.6)	
IGCCCG grouping (n, % within IGCCCG)			0.1097
Good (*n* = 45)	5 (11.1)	40 (88.9)	
Intermediate/Poor (*n* = 16)	5 (31.3)	11 (68.7)	

Abbreviations: IC—immune cells; IGCCCG—International Germ Cell Cancer Collaborative Group; LVI—lymphovascular invasion. Row percentages are depicted.

**Table 3:** Survival impact of PD-L1 immunoexpression in immune cells in univariable and multivariable analyses.

Relapse-Free Survival	
**Variables ^#^**	**Univariable**
PD-L1 expression in ICs	HR = 4.481, 95%CI 1.366–14.697, *p* = 0.013 *
	**Multivariable (PD-L1 expression in ICs, adjusted for…)**
Histology (SE vs NS)	HR = 4.198, 95%CI 1.268–13.899, *p* = 0.019 *
LVI	HR = 4.647, 95%CI 1.380–15.651, *p* = 0.013 *
pT Stage	HR = 4.683, 95%CI 1.397–15.700, *p* = 0.012 *
N Stage	HR = 3.938, 95%CI 1.108–13.996, *p* = 0.034 *
Stage (TNM)	HR = 4.209, 95%CI 1.275–13.896, *p* = 0.018 *
IGCCCG grouping	HR = 5.398, 95%CI 0.902–32.302, *p* = 0.065

* Significant values. # Reference categories: PD-L1 positive ICs; SE; no LVI; pT1 tumors; N0 tumors; stage I tumors; Good prognosis category. Abbreviations: CI—confidence interval; HR—hazard ratio; IC—immune cells; IGCCCG—International Germ Cell Cancer Collaborative Group; LVI—lymphovascular invasion; NS—non-seminoma; SE—seminoma.

**Table 4:** Association between CD20 and CD3 immunoexpression in immune cells populating seminomas and clinicopathological features.

Variables	CD20 in ICs: Low	CD20 in ICs: High	*p*-value
LVI (*n*, % within LVI)			0.6537
Yes (*n* = 31)	17 (54.8)	14 (45.2)	
No (*n* = 55)	26 (47.3)	29 (52.7)	
*Rete testis* invasion (*n*, % within *rete testis* invasion)			0.0079*
Yes (*n* = 46)	29 (63.0)	17 (37.0)	
No (*n* = 37)	12 (32.4)	25 (67.6)	
pT Stage (*n*, % within pT Stage)			0.1949
pT1 (*n* = 45)	19 (42.2)	26 (57.8)	
pT2-3 (*n* = 41)	24 (58.5)	17 (41.5)	
N Stage (*n*, % within N Stage)			0.3155
N0 (*n* = 65)	30 (46.2)	35 (53.8)	
N+ (*n* = 20)	12 (60.0)	8 (40.0)	
Stage, TNM (*n*, % within Stage, TNM)			0.0216*
I (*n* = 65)	30 (46.2)	35 (53.8)	
II (*n* = 14)	6 (42.9)	8 (57.1)	
III (*n* = 7)	7 (100)	0 (0)	
IGCCCG grouping (*n*, % within IGCCCG)			0.5048
Good (*n* = 19)	11 (57.9)	8 (42.1)	
Intermediate/Poor (*n* = 2)	2 (100)	0 (0)	
	**CD3 in ICs: Low**	**CD3 in ICs: High**	***p*-Value**
LVI (*n*, % within LVI)			0.3353
Yes (*n* = 31)	12 (38.7)	19 (61.3)	
No (*n* = 55)	15 (27.3)	40 (72.7)	
pT Stage (*n*, % within pT Stage)			0.0211*
pT1 (*n* = 45)	9 (20.0)	36 (80.0)	
pT2-3 (*n* = 41)	18 (43.9)	23 (56.1)	
N Stage (n, % within N Stage)			0.4054
N0 (*n* = 65)	18 (27.7)	47 (72.3)	
N+ (*n* = 20)	8 (40.0)	12 (60.0)	
Stage, TNM (*n*, % within Stage TNM)			0.0291*
I–II (*n* = 79)	22 (27.8)	57 (72.2)	
III (*n* = 7)	5 (71.4)	2 (28.6)	
IGCCCG grouping (*n*, % IGCCCG)			0.1714
Good (*n* = 19)	7 (36.8)	12 (63.2)	
Intermediate/Poor (*n* = 2)	2 (100)	0 (0)	

Abbreviations: IC—immune cells; IGCCCG—International Germ Cell Cancer Collaborative Group; LVI—lymphovascular invasion. Row percentages are depicted.

**Table 5:** Immunoexpression of CTLA-4, PD-L1 and MMR proteins in tumor cells of testicular germ cell tumors.

Variables	CTLA-4 in TCs: Absent/Weak	CTLA-4 in TCs: Moderate/Strong	*p*-Value
Primary tumor histology (*n*, % within Histology)			<0.0001 *
SE (*n* = 107)	74 (69.2)	33 (30.8)	
NS (*n* = 157)	58 (36.9)	99 (63.1)	
Primary tumor subtype (*n*, % within subtype)			
SE (*n* = 107)	74 (69.2)	33 (30.8)	<0.0001 *
EC (*n* = 60)	41 (68.3)	19 (31.7)	
YST (*n* = 39)	10 (25.6)	29 (74.4)	
CH (*n* = 11)	1 (9.1)	10 (90.9)	
TE (*n* = 47)	6 (12.8)	41 (87.2)	
	**PD-L1 in TCs: Negative**	**PD-L1 in TCs: Positive**	***p*-Value**
Histology (*n*, % within Histology)			1.0
SE (*n* = 108)	81 (75.0)	27 (25.0)	
NS (*n* = 157)	118 (75.2)	39 (24.8)	
Primary tumor subtype (*n*, % within subtype)			
SE (*n* = 108)	81 (75.0)	27 (25.0)	<0.0001*
EC (*n* = 60)	38 (63.3)	22 (36.7)	
YST (*n* = 39)	33 (84.6)	6 (15.4)	
CH (*n* = 11)	1 (9.1)	10 (90.9)	
TE (*n* = 47)	46 (97.9)	1 (2.1)	
	**MLH1 in TCs: CS<6**	**MLH1 in TCs: CS≥6**	***p*-Value**
Histology (*n*, % within Histology)			<0.0001*
SE (*n* = 108)	18 (16.7)	90 (83.3)	
NS (*n* = 154)	82 (53.2)	72 (46.8)	
Primary tumor subtype (*n*, % within subtype)			
SE (*n* = 108)	18 (16.7)	90 (83.3)	<0.0001*
EC (*n* = 59)	26 (44.1)	33 (55.9)	
YST (*n* = 39)	18 (46.2)	21 (53.8)	
CH (*n* = 11)	6 (54.5)	5 (45.5)	
TE (*n* = 45)	32 (71.1)	13 (28.9)	
	**PMS2 in TCs: CS<6**	**PMS2 in TCs: CS≥6**	***p*-Value**
Histology (*n*, % within Histology)			0.0001*
SE (*n* = 107)	16 (15.0)	91 (85.0)	
NS (*n* = 154)	56 (36.4)	98 (63.6)	
Primary tumor subtype (*n*, % within subtype)			
SE (*n* = 107)	16 (15.0)	91 (85.0)	0.0003*
EC (*n* = 59)	18 (30.5)	41 (69.5)	
YST (*n* = 39)	12 (30.8)	27 (69.2)	
CH (*n* = 11)	3 (27.3)	8 (72.7)	
TE (*n* = 45)	23 (51.1)	22 (48.9)	
	**MSH2 in TCs: CS<6**	**MSH2 in TCs: CS≥6**	***p*-Value**
Histology (*n*, % within Histology)			0.8287
SE (*n* = 108)	9 (8.3)	99 (91.7)	
NS (*n* = 154)	15 (9.7)	139 (90.3)	
Primary tumor subtype (n, % within subtype)			
SE (*n* = 108)	9 (8.3)	99 (91.7)	0.0005*
EC (*n* = 59)	0 (0)	59 (100)	
YST (*n* = 39)	3 (7.7)	36 (92.3)	
CH (*n* = 11)	4 (36.4)	7 (63.6)	
TE (*n* = 45)	8 (17.8)	37 (82.2)	
	**MSH6 in TCs: CS<6**	**MSH6 in TCs: CS≥6**	***p*-Value**
Histology (*n*, % within Histology)			0.0865
SE (*n* = 107)	12 (11.2)	95 (88.8)	
NS (*n* = 150)	29 (19.3)	121 (80.7)	
Primary tumor subtype (*n*, % within subtype)			
SE (*n* = 107)	12 (11.2)	95 (88.8)	<0.0001 *
EC (*n* = 58)	1 (1.7)	57 (98.3)	
YST (*n* = 38)	8 (21.1)	30 (78.9)	
CH (*n* = 10)	5 (50.0)	5 (50.0)	
TE (*n* = 44)	15 (34.1)	29 (65.9)	

* significant values. Abbreviations: CH—choriocarcinoma; CS—combined score; EC—embryonal carcinoma; MMR – mismatch repair; NS—non-seminoma; SE—seminoma; TC—tumor cells; TE—postpubertal-type teratoma; YST—postpubertal-type yolk sac tumor. Row percentages are depicted.

## Supplementary Materials

The following are available online at https://www.mdpi.com/2072-6694/11/10/1535/s1, Figure S1: Immunoexpression of CTLA-4 in immune cells of testicular germ cell tumors and association with prognostic features, Figure S2: Relapse-free survival according to CTLA-4 immunoexpression in immune cells of testicular germ cell tumors, Figure S3: Characterization of the immune infiltrate populating pure seminomas, Figure S4: Illustrative examples of characteristics of the immune infiltrate in pure seminomas, Figure S5: Absent and weak immunoexpression of CTLA-4 in tumor cells, Figure S6: Relapse-free survival according to CTLA-4 immunoexpression intensity in tumor cells of testicular germ cell tumors, Figure S7: Relapse-free survival according to PD-L1 immunoexpression in tumor cells of testicular germ cell tumors, Figure S8: Immunoexpression of PD-L1 and CTLA-4 among primary and metastatic testicular germ cell tumor samples, Figure S9: Immunoexpression of mismatch repair proteins in metastatic testicular germ cell tumor samples. Combined score for MLH1 (A), PMS2 (B), MSH2 (C) and MSH6 (D) among residual mature teratoma and non-teratoma viable disease, Figure S10: mRNA expression of CTLA-4 in the TCGA database for Testicular Germ Cell Tumors. Table S1: Mixed Testicular Germ Cell Tumors and proportions of each component, Table S2: Immunohistochemistry methodology details. Table S3: Raw data related to immunoscoring of primary tumor samples, Table S4: Raw data related to immunoscoring of metastatic samples.
