# Supplementary material for: Detailed Characterization of Immune Cell Infiltrate and Expression of Immune Checkpoint Molecules PD-L1/CTLA-4 and MMR Proteins in Testicular Germ Cell Tumors Disclose Novel Disease Biomarkers
Source: Cancers (Basel). 2019 Oct 11;11(10):1535. doi: 10.3390/cancers11101535 (PMC6826711; doi:10.3390/cancers11101535)
Supplement: Supplementary file 1 [file cancers-11-01535-s001.zip › cancers-583339-SUPPL.pdf]

Supplementary Materials:

Detailed Characterization of Immune Cell Infiltrate and Expression of Immune Checkpoint Molecules PD-L1/CTLA-4 and MMR Proteins in Testicular Germ Cell Tumors Disclose Novel Disease Biomarkers

João Lobo, Ângelo Rodrigues, Rita Guimarães, Mariana Cantante, Paula Lopes, Joaquina Maurício, Jorge Oliveira, Carmen Jerónimo and Rui Henrique

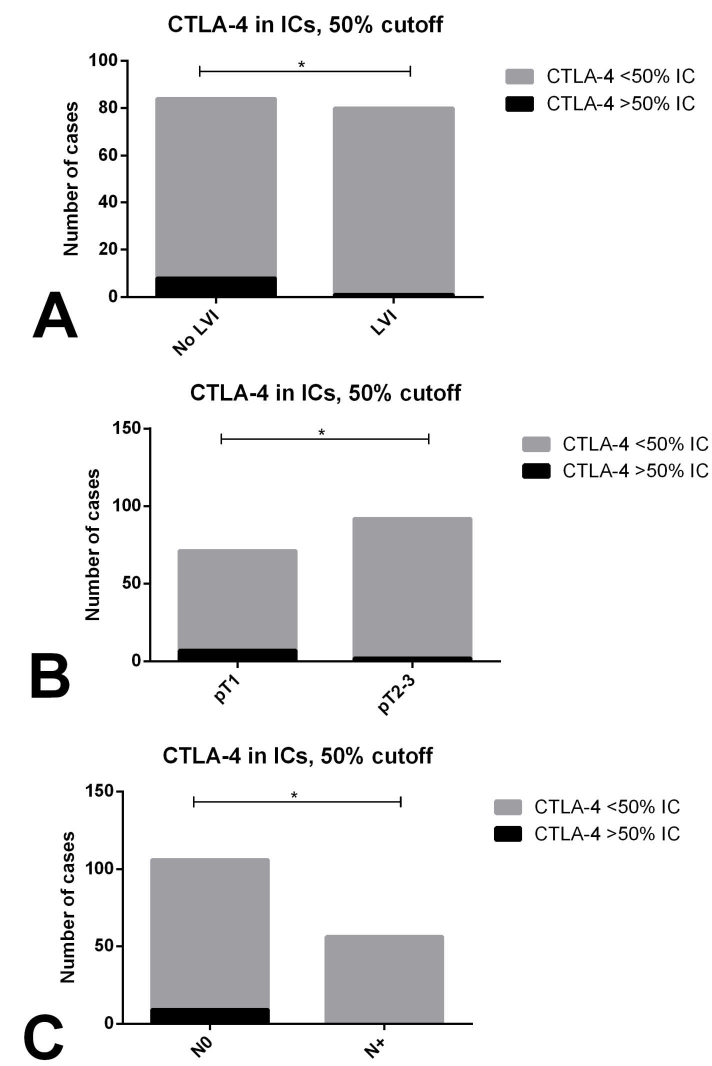

Figure S1. Immunoexpression of CTLA-4 in immune cells of testicular germ cell tumors and association with prognostic features. Association between CTLA-4 immunoexpression above the 50% cutoff in immune cells and lymphovascular invasion (**A**), pT stage (**B**) and N stage (**C**). Abbreviations: LVI—lymphovascular invasion; IC—immune cells. * refers to $p < 0.05$.

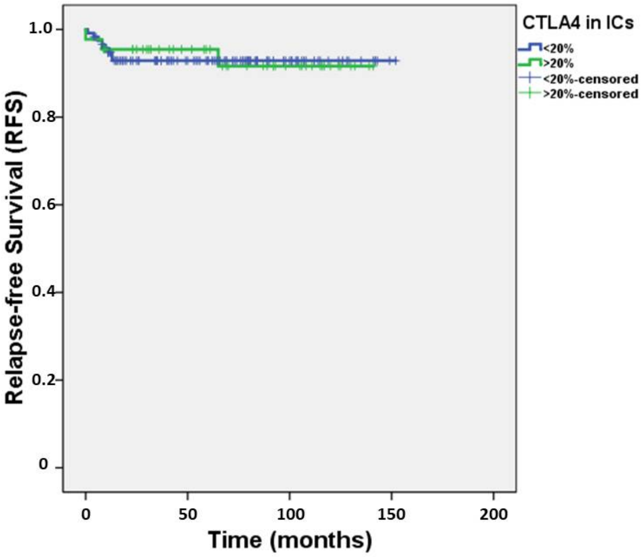

Figure S2. Relapse-free survival according to CTLA-4 immunoexpression in immune cells of testicular germ cell tumors. Abbreviations: IC—immune cells; RFS—relapse-free survival.

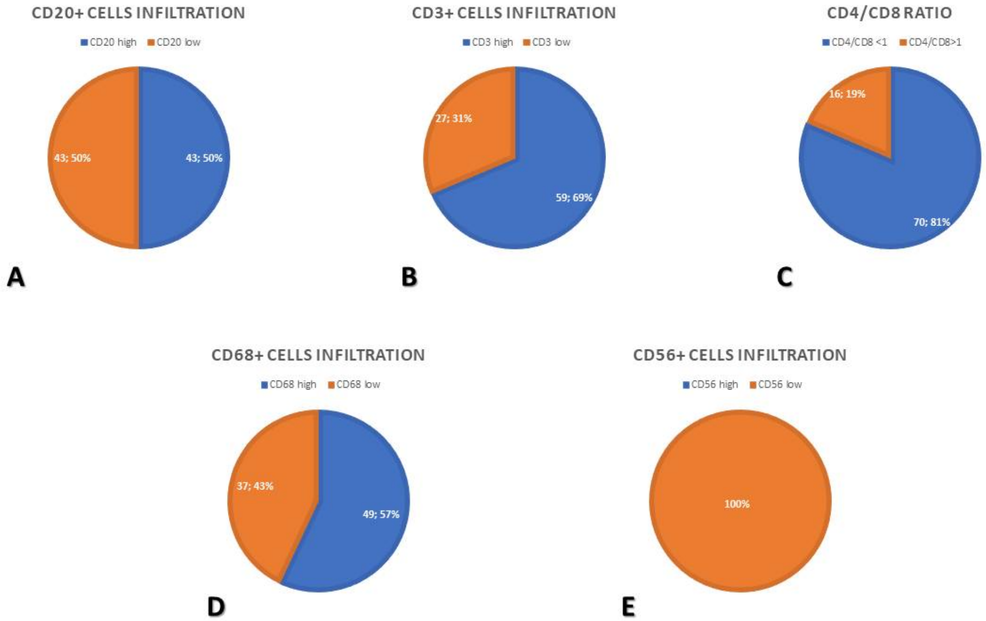

Figure S3. Characterization of the immune infiltrate populating pure seminomas. Categorization of tumors according to the amount of CD20-positive B-lymphocytes (**A**), CD3-positive T-lymphocytes (**B**), the CD4/CD8 ratio (**C**), CD68-positive macrophages (**D**) and CD56-positive natural killer cells (**E**).

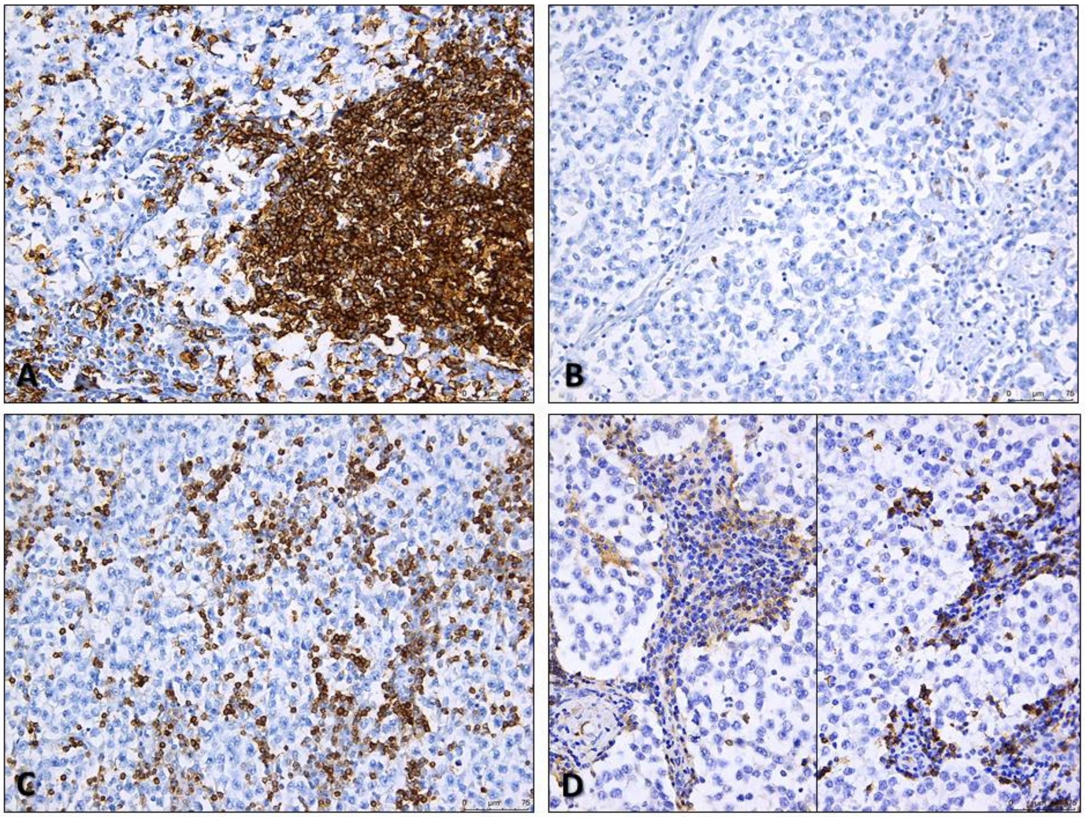

Figure S4. Illustrative examples of characteristics of the immune infiltrate in pure seminomas. **A**—Pure seminoma with abundant CD20-positive B-lymphocyte infiltration, both peripheral and intratumoral, forming follicles across the whole representative slide (200×) and **B**—Pure seminoma with very few CD20-positive B-cells within the fibrous septa across the whole slide (200×); **C**—Pure seminoma showing a high infiltration with CD3-positive T-lymphocytes (200×); **D**—Pure seminoma showing presence of both CD4-positive (left) and CD8-positive (right) T-lymphocytes (200×).

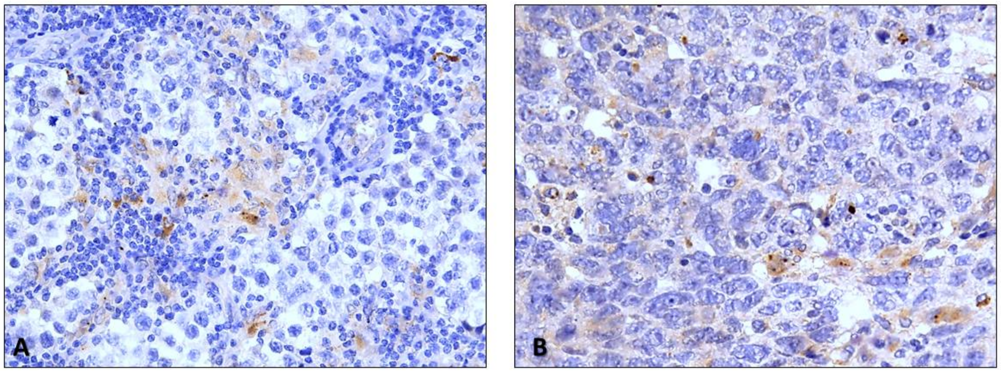

Figure S5. Absent and weak immunoexpression of CTLA-4 in tumor cells. **A**—Pure seminoma showing absence of specific staining with CTLA-4 in the tumor cells; **B**—Pure embryonal carcinoma showing very faint cytoplasmic staining for CTLA-4 in tumor cells, that was only depicted in high power (400×).

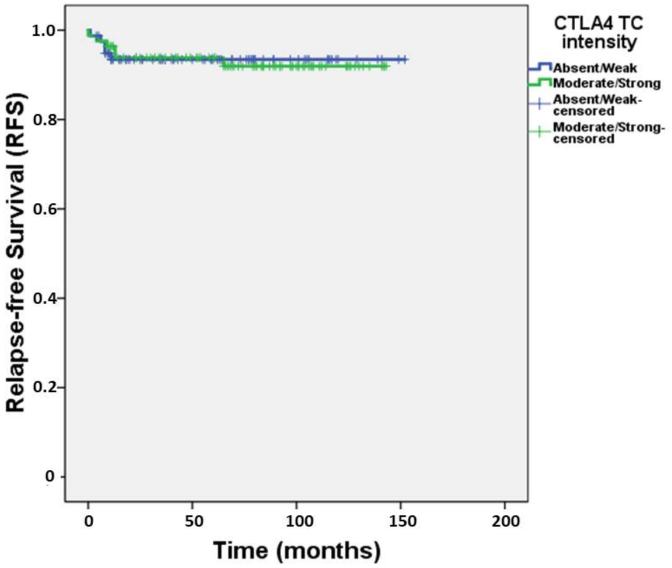

Figure S6. Relapse-free survival according to CTLA-4 immunoexpression intensity in tumor cells of testicular germ cell tumors.

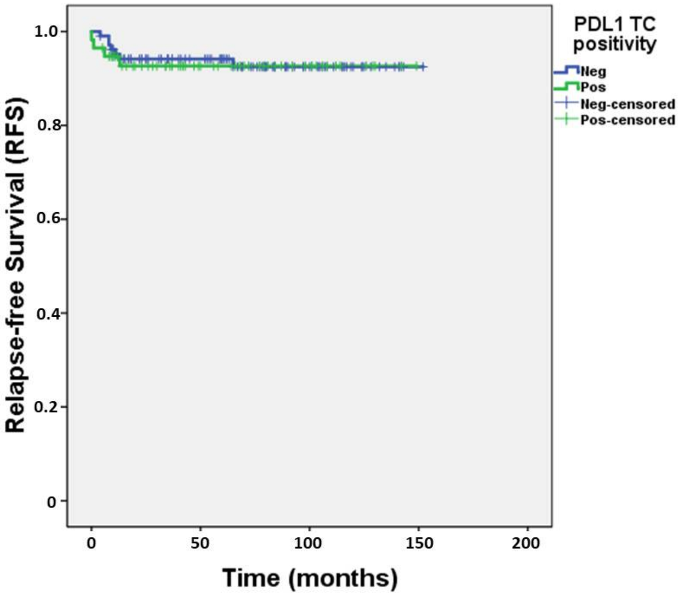

Figure S7. Relapse-free survival according to PD-L1 immunoexpression in tumor cells of testicular germ cell tumors.

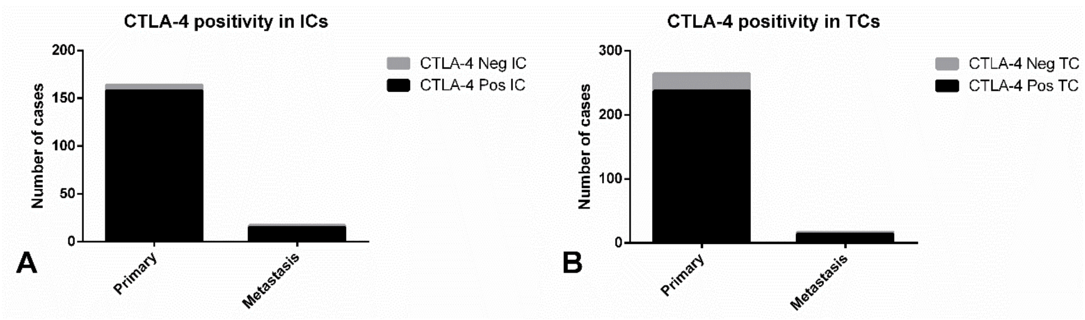

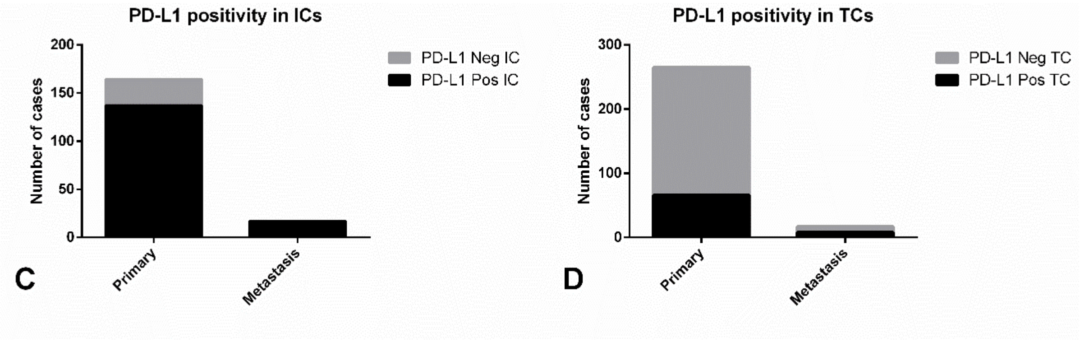

Figure S8. Immunoexpression of PD-L1 and CTLA-4 among primary and metastatic testicular germ cell tumor samples, **A** and **C**—Positivity in immune cells among primary and metastatic tumors; **B** and **D**—Positivity in tumor cells among primary and metastatic tumors.

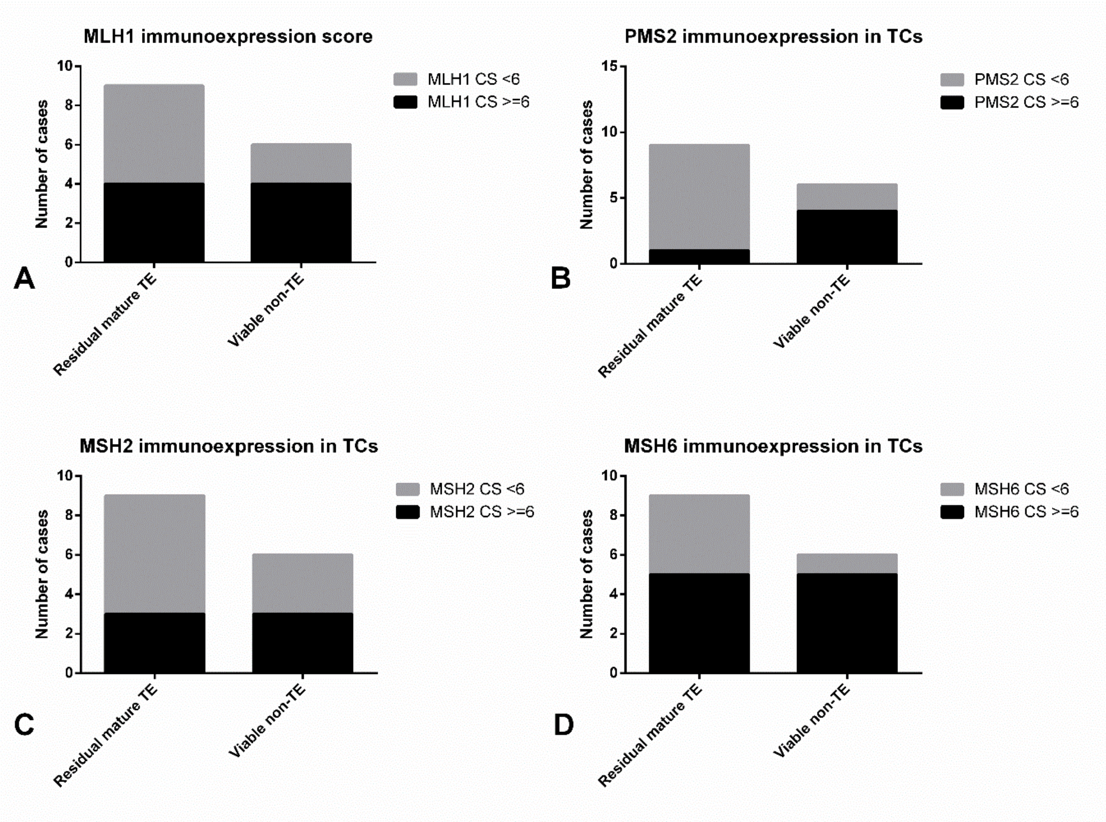

Figure 9. Immunoexpression of mismatch repair proteins in metastatic testicular germ cell tumor samples. Combined score for MLH1 (**A**), PMS2 (**B**), MSH2 (**C**) and MSH6 (**D**) among residual mature teratoma and non-teratoma viable disease.

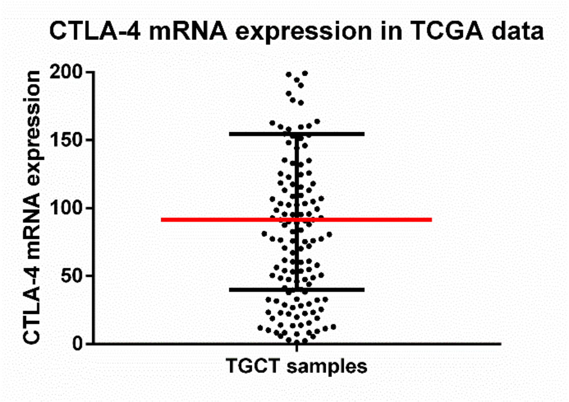

Figure S10. mRNA expression of CTLA-4 in the TCGA database for Testicular Germ Cell Tumors. TGCT—testicular germ cell tumors.

Tables S1–S4 are in a separately file

© 2019 by the authors. Licensee MDPI, Basel, Switzerland. This article is an open access article distributed under the terms and conditions of the Creative Commons Attribution (CC BY) license (http://creativecommons.org/licenses/by/4.0/).
